# Subzero Temperature Storage to Preserve the Quality Attributes of Veiled Virgin Olive Oil

**DOI:** 10.3390/foods12061228

**Published:** 2023-03-13

**Authors:** Anna Díez-Betriu, Agustí Romero, Antonia Ninot, Alba Tres, Stefania Vichi, Francesc Guardiola

**Affiliations:** 1 Departament de Nutrició, Ciències de l’Alimentació i Gastronomia, Campus de l’Alimentació de Torribera, Facultat de Farmàcia i Ciències de l’Alimentació, Universitat de Barcelona, 08921 Santa Coloma de Gramenet, Spain; annadiez@ub.edu (A.D.-B.); atres@ub.edu (A.T.); fguardiola@ub.edu (F.G.); 2 Institut de Recerca en Nutrició i Seguretat Alimentària (INSA-UB), Universitat de Barcelona (UB), 08921 Santa Coloma de Gramenet, Spain; 3 Institute of Agrifood Research and Technology (IRTA), Mas Bové Ctra. Reus-El Morell km 3.8, 43120 Constantí, Spain; agusti.romero@irta.cat (A.R.); antonia.ninot@irta.cat (A.N.)

**Keywords:** veiled olive oil, filtration, cold storage, frozen storage, freezing speed, quality parameters, sensory quality

## Abstract

Unfiltered olive oils (UO) have gained popularity in the global market, but they lose their quality characteristics faster than filtered oils (FO). In this work, refrigeration and freezing temperatures were explored to maintain UO quality features during storage. A full factorial design was applied to an UO and to the same oil after filtration to evaluate the effect of storage temperature (room temperature, 4 °C and –20 °C) and freezing speed (slow-freezing, in the freezer at −20 °C and fast-freezing, in a bath of liquid nitrogen). Official quality parameters, polar and nonpolar phenolic compounds, oxidative stability index, volatile compounds and descriptive sensory profile were measured periodically over 24 months of storage in the dark. Storage temperature influenced the quality of both UO and FO, but in different ways. At non-freezing temperature, UO experienced a severe decrease in its sensory quality compared to FO, mainly due to the hydrolysis of secoiridoids and degradation of the volatile fraction, but storage at −20 °C allowed to effectively preserve UO quality traits, thus resulting as a suitable strategy to increase the shelf-life of UO to satisfy the demand of consumers for this particular product. The results showed that slow-freezing was the most appropriate method for freezing.

## 1. Introduction

Extra virgin olive oil (EVOO) typically undergoes a filtration step before bottling and commercialization to remove any suspended solids and residual vegetation water that could negatively impact its stability during storage [1,2]. However, some EVOO is sold without filtering [1] to satisfy the increasing demand from some consumers, who perceive unfiltered oils (UO) as less processed and appreciate their particular organoleptic properties [3]. UO, also known as veiled EVOO, has a cloudy appearance due to the presence of micro-droplets of vegetation water and solid dispersed particles from the olive [4], which would otherwise be removed during the filtration step [2]. Filtration is, in fact, one of the most controversial steps in olive oil production [1]. During filtration, quantitative and qualitative changes take place, especially in minor components [2]. Filtration may result in partial removal of polar phenolic compounds present in the vegetation water [2] and a loss of volatile compounds [5], which can negatively impact the organoleptic profile of the oil.

Polar phenolic compounds are of particular significance for their influence on the sensory quality and oxidative stability of the oil [6,7], as well as for their potential beneficial effects on human health [8]. Their concentration in EVOO depends on several factors including geographic origin, olive variety and maturity and processing methods, and can range from 0.02 to 600 mg/kg [9]. Secoiridoid derivatives (SEC) are the most important class of polar phenolic compounds in EVOO, and they can be divided into oleuropein (OL) and ligstroside (LIG) derivatives, which are chemically characterized by a phenyl ethyl alcohol (hydroxytyrosol (HTy) in OL and tyrosol (Ty) in LIG) linked to elenolic acid or its derivatives. The volatile compounds responsible for the desired aroma of EVOO are mainly C5 and C6 aldehydes, alcohols and ketones originating from the enzymatic oxidation of fatty acids through the lipoxygenase (LOX) pathway [10]. In high-quality EVOO, LOX compounds make up 60–80% of the volatile fraction [11].

Different authors have reported contradictory information on the effect of filtration on EVOO quality and stability. On the one hand, filtration has been reported to decrease oxidative stability due to increased exposure to oxygen and the depletion of polar phenols. However, the differences in oxidative stability between filtered oil (FO) and UO are sometimes minimal [1,12,13]. On the other hand, the presence of water and microorganisms in UO can lead to a rapid deterioration of UO during storage. The moisture content in UO can be as high as 1.5% (*w*/*w*), exceeding the maximum recommended level of 0.2%, set by the IOC to prevent the formation of off-flavors [14]. The high water content promotes rancidity and hydrolytic reactions [2]. A study by Fregapane et al. [12] found that during storage, UO underwent a higher rate of hydrolysis of triacylglycerols (TAG) and SEC than FO. On the one side, the hydrolysis of TAG results in an increase in free fatty acids (FFA) of the oil. On the other side, hydrolysis of SEC takes place at the ester bond between the phenyl ethyl alcohol and the elenolic acid or its derivates, thus reducing the content of SEC aglycons and increasing the content of HTy and Ty.

The water activity in UO may range from 0.5 to more than 0.8 [12,15]. This promotes the proliferation of spoiling bacteria, yeasts and molds that are found in higher amounts in UO [16,17], leading to the development of fermentative sensory defects such as fusty, musty-humid, muddy and winey-vinegary, even during the early stages of UO storage [18,19].

In this regard, storage conditions may play a significant role in the preservation of UO, as it is more prone to hydrolytic, enzymatic and microbiological degradation compared to FO. Hence, in the recent years, there have been a growing number of studies focused on evaluating the stability of UO during storage [16,20,21,22,23,24,25,26,27,28,29]. Several efforts have been made to extend the shelf life of UO by applying techniques that are unconventional for the processing and storage of standard FO, such as high hydrostatic pressure [19] and modified atmosphere [24,26,27]. The use of techniques that incur higher storage costs is justified by the demand for a unique product such as UO, which can command higher prices and appeal to consumers.

To date, the use of cold storage to enhance the shelf life of UO has been scarcely investigated [30], despite evidence of its positive impact on FO quality, as seen in studies on refrigeration [31,32,33] and freezing temperatures [30,34,35,36,37]. The application of low temperatures to the storage of UO is expected to decrease the rate of chemical reactions that cause hydrolytic degradation. Subzero temperature storage may be particularly effective in reducing the growth of spoilage microorganisms and preserving the sensory characteristics of the oil. Further research is necessary to assess the effect of refrigerated and frozen storage on the chemical and sensory quality of UO. The best freezing method should also be assessed, given that at low temperatures the oil might crystallize, leading to different solid/liquid ratios depending on the temperature. While some authors [38] reported higher oxidation rates during the phase transition due to the increase in unsaturated TAG and the decrease in polar phenols in the liquid phase surrounding fat crystals [39], other authors have observed small differences in the oxidation rates between the solid and liquid phases [40], and have found a higher content of α-tocopherol and polar phenols in the liquid phase. It would be necessary to compare the effects of conventional freezing methods and faster methods, such as the use of liquid nitrogen, to evaluate the impact of selective crystallization of TAG and partitioning of phenols between liquid and solid phases on the degradation rate of UO.

The purpose of this study was to assess storage conditions suitable for preserving the quality of UO. Quality and composition parameters of an UO were monitored under different storage conditions for 24 months, and the changes were compared to the changes in quality and composition parameters of the same oil after its filtration. A full factorial design was applied to evaluate the impact of storage temperature (room temperature (RT), 4 °C (R) and −20 °C) and freezing speed (slow-freezing (S), by placing the oil at −20 °C, or fast-freezing (F), by immersing the oil in a liquid nitrogen bath at −196 °C) on the oil’s quality. Additionally, the effect of thawing oils that had been stored at −20 °C for 12 months, and their further storage at RT was evaluated. The oils were examined for trade quality parameters, phenols (tocopherols and polar phenolic compounds), oxidative stability (measured as Oxidative Stability Index, OSI), volatile compounds and through descriptive sensory assessment.

## 2. Materials and Methods

### 2.1. Samples

Two EVOO from the Picual cultivar were sourced from Castillo de Canena (Canena, Jaén, Spain). Both oils were produced on the same day using the same batch of olives. Oil extraction was performed using a two-phase decanter. One of the oils was filtered by the producer using cellulose filters (FO), while the other was left unfiltered (UO).

### 2.2. Storage Conditions

The two oils were homogenized, and were each portioned into 100 mL aliquots, which were stored in 130 mL glass bottles from Scharlau (Sentmenat, Spain) made of borosilicate glass 3.3 with high density polypropylene caps. Samples were kept in the dark for 24 months under different storage conditions, including three temperatures: 20–25 °C (RT), 4 °C (R) and −20 °C. Moreover, two freezing methods were used for the samples stored at −20 °C: a fast-freezing method (F), achieved by immersing the samples in a bath of liquid nitrogen, and a slow-freezing method (S), performed by placing the samples directly in a freezer set to −20 °C. The experimental design, resumed in Figure S1a of Supplementary Materials, was replicated twice.

After initial characterization of the oils, the samples were analyzed at 6, 12 and 24 months. Before analysis, refrigerated and frozen samples were tempered and thawed by placement at RT in the dark.

In addition, FO and UO samples of the S frozen condition were thawed at 12 months (TH) and analyzed at two time points: right after thawing (THT_0_) and after 6 months of storage at RT (THT_6_). These samples were compared with the same FO and UO samples (control) analyzed at the beginning of the conservation study (CT_0_) and after 6 months of storage at RT (CT_6_) (Supplementary Materials, Figure S1b), with the aim to verify if olive oil experiences modifications that could result in a faster decline in quality after thawing, as some authors have hypothesized [30,36].

A total of 8 aliquots were prepared for each oil, time and storage condition, so that determinations of each oxidative parameter could be carried out from a freshly opened bottle in each point of analysis.

### 2.3. Analytical Methods

#### 2.3.1. Trade Quality Indices

The determinations of free fatty acids (FFA), peroxide value (PV) and extinction coefficients (K_232_ and K_268_) were conducted following the analytical methods outlined in the Commission Regulation (EEC) No. 2568/91 and subsequent amendments [41].

The sensory analysis was carried out following the method described in Commission Regulation (EEC) No. 2568/91 and subsequent amendments [41] by the Official Tasting Panel of Virgin Olive Oils of Catalonia, which follows ISO 17025 and the International Olive Council (IOC) accreditation standards. Intensity of positive attributes and sensory defects were assessed and expressed as a median of the panelists’ scores. Moreover, an open generic profile was used to identify secondary sensory attributes, expressed as percentage of panelists who were able to detect each odor note [42].

#### 2.3.2. Moisture and Volatile Matter

Determination of moisture content and volatile matter was carried out according to the AOCS official method Ca 2d-25 [43] using the vacuum oven method.

#### 2.3.3. Oxidative Stability Index (OSI)

OSI was determined according to the AOCS official method Cd 12b-92 [44] under the following conditions: a temperature of 120 °C, air flow rate of 20 L/h using an 892 Professional Rancimat (Metrohm, Herisau, Switzerland).

#### 2.3.4. Fatty Acids

A double methylation was performed to prepare the fatty acid methyl esters (FAME), which were then separated by gas chromatography-flame ionization detector (GC-FID) as described by Díez-Betriu et al. [37]. Briefly, a 1μL sample of FAME extract was injected into an Agilent 4890D gas chromatograph (Agilent Technologies, Santa Clara, CA, USA), equipped with a flame ionization detector (FID) and a split-splitless injector. Separation was achieved with a SP-2380 column from Supelco Ltd., Bellefonte, PA, USA (60 m × 0.25 mm and 0.2 μm film thickness). The oven temperature was as increased as follows: 1 min at 150 °C, from 150 to 180 °C at 1.5 °C/min, 0.5 min at 180 °C, from 180 to 220 °C at 14.5 °C/min, 3 min at 220 °C, from 220 to 250 °C at 9.9 °C/min, and 9 min at 250 °C. The carrier gas was hydrogen at 25 psi, with a split ratio of 1:30. The injector temperature was set at 270 °C and the detector temperature at 300 °C. Each compound was identified by comparing its retention time with that of standards (Supelco 37 component FAME Mix, Supelco^®^, Merck KGaA, Darmstadt, Germany). The percentage of each fatty acid was calculated using peak area normalization.

#### 2.3.5. Polar and Lipophilic Phenolic Compounds

Polar phenolic compounds were extracted following the method described by Vichi et al. [45] and analyzed by ultra-high performance liquid chromatography-diode array detector (UHPLC-DAD), adapting the chromatographic conditions of the IOC method COI/T.29/Doc No 29 [46] to an UHPLC system, as described by the same authors in a previous study [47]. Briefly, the phenolic extract (15 μL) was injected into an Acquity-UPLC (Waters, Milford, MA, USA) coupled to a PDA 2996 detector (Waters, Milford, MA, USA). Separation was achieved by using a Halo C18 Fused-Core column (2.1 mm × 100 mm and particle size of 2.7 μm) from Advanced Materials Technology (Wilmington, DE, USA). Elution was conducted at a flow rate of 0.4 mL/min and 30 °C, using a mobile phase consisting of ultrapure water (Milli-Q Millipore Corporation, Billerica, MA, USA) and formic acid (98:2, *v*/*v*) as solvent A, and a mixture of methanol and acetonitrile (50:50, *v*/*v*) as solvent B. The solvent gradient changed according to the following conditions: from 96% (A) : 4% (B), to 20% (B) at 5 min, to 45% (B) at 28 min, to 100% (B) at 30 min, held at 100% (B) for 5 min, then 96% (A) : 4% (B) at 36 min, followed by 5 min of equilibration. Detection was carried out simultaneously at 335 nm and 280 nm. Identification of the compounds was performed according to Mateos et al. [48] and the IOC method COI/T.29/Doc No 29 [46], and was further confirmed by high resolution mass spectrometry using a Q-Exactive hybrid Orbitrap (Thermo Fisher Scientific, Bremen, Germany) under the same chromatographic conditions. The settings for the spectrometer and ion source were according to Vichi et al. [45]. The dialdehydic form of LIG aglycone and one of the oxidized aldehyde and hydroxylic forms of OL aglycone could not be quantified due to coelution by UHPLC-DAD analysis. Polar phenolic compounds were quantified using response factors from Mateos et al. [48]. The internal standard (IS) used for flavones was *o*-coumaric acid, while, for the rest of the compounds, the IS was *p*-hydroxyphenylacetic acid. The results were expressed in mg/kg.

Tocopherols were measured according to the AOCS official method Ce 8–89 [49] with some modifications. The sample was weighed (1.5 g) and diluted in a 10 mL volumetric flask with n-hexane. After filtration, 20 μL of the solution were injected into an Agilent 1100 series HPLC (Agilent Technologies, Santa Clara, CA, USA) coupled to a Hewlett-Packard 1046A fluorescent detector. Separation was performed using a 4 × 3.0 mm precolumn (Phenomenex Security Guard Cartridge Silica) and a Luna silica column (150 × 4.6 mm i.d., 3 μm particle size and 100 Å pore size) from Phenomenex (Torrance, CA, USA). Elution was carried out using n-hexane/1,4-dioxane (95/5% *v*/*v*) as the mobile phase.

#### 2.3.6. Volatile Compounds

The extraction of the volatile compounds was conducted through headspace solid-phase microextraction (HS-SPME) with some modification to the method described by Vichi et al. [50]: 2 g of the sample were conditioned during 10 min in a silicon bath at 40 °C under magnetic stirring. The volatile compounds were analyzed using a gas chromatography–mass spectrometry (GC–MS) system consisting of an Agilent GC 6890N with a split-splitless injector and a quadrupole mass selective spectrometer 5973, both from Agilent Technology, Palo Alto, CA, USA. The separation of the volatile compounds was achieved using a Supelcowax-10 column (60 m × 0.25 mm i.d., 0.25 μm film thickness), purchased from Supelco Ltd. (Bellefonte, PA, USA). The GC oven program was as follows: 10 min at 40 °C, increased at 3 °C/min up to 150 °C, then at 15 °C/min up to 250 °C and at 250 °C for 5 min. Helium was the carrier gas, with 1.5 mL/min of flow rate. The transfer line and ion source temperatures were 275 °C and 200 °C, respectively. The recording of electron impact mass spectra was performed at 70 eV ionization energy in the *m/z* range 35–300, 2 scan/s. The volatile compounds were identified by comparison with reference compounds and by comparing their mass spectral data with those of the Wiley 6 mass spectra library. Semi-quantification was conducted by spiking the samples with 10 μL of IS (4-methyl-2-pentanol, 0.2 mg/mL sunflower oil). The results were expressed as mg of 4-methyl-2-pentanol per kg of oil.

### 2.4. Statistical Analysis

The effects of the storage conditions (storage temperature and freezing method) and time of storage on the measured parameters were evaluated in FO and UO separately by means of two-way ANOVA (*n* = 32; 4 storage conditions × 4 storage times × 2 experimental replicates). Moreover, a multifactor ANOVA (*n* = 64; 2 oils × 4 storage conditions × 4 storage times × 2 experimental replicates) was applied to assess the effect of filtration, that is, whether the effect of the storage conditions was significantly different depending on filtration.

In order to verify whether olive oil undergoes a faster loss of quality after thawing (TH), two-way ANOVA was applied to FO and to UO control and TH samples (*n* = 8; control and TH samples × 2 storage times (T_0_ and T_6_) × 2 experimental replicates).

In all the cases, SPSS Statistics (v.26, IBM, Armonk, NY, USA) was used. *p*-Values lower than or equal to 0.05 were considered significant.

## 3. Results and Discussion

Trade quality indices, phenolic and volatile compounds, and sensory attributes (including both positive and negative notes and secondary attributes) of UO were monitored under different storage conditions such as RT, R and subzero temperature (F or S freezing method). To assess the impact of these storage conditions on the stability of UO, compared with a conventional FO, the same EVOO was filtered and submitted to the same storage conditions and analytical determinations.

### 3.1. Trade Quality Indices and Oxidative Stability Index (OSI)

As previously observed in other studies [13], the initial values of trade quality indices for both UO and FO were comparable, meeting the criteria for the “extra virgin” category [41] based on their physical and chemical parameters (Table 1).

**Figure 1:**
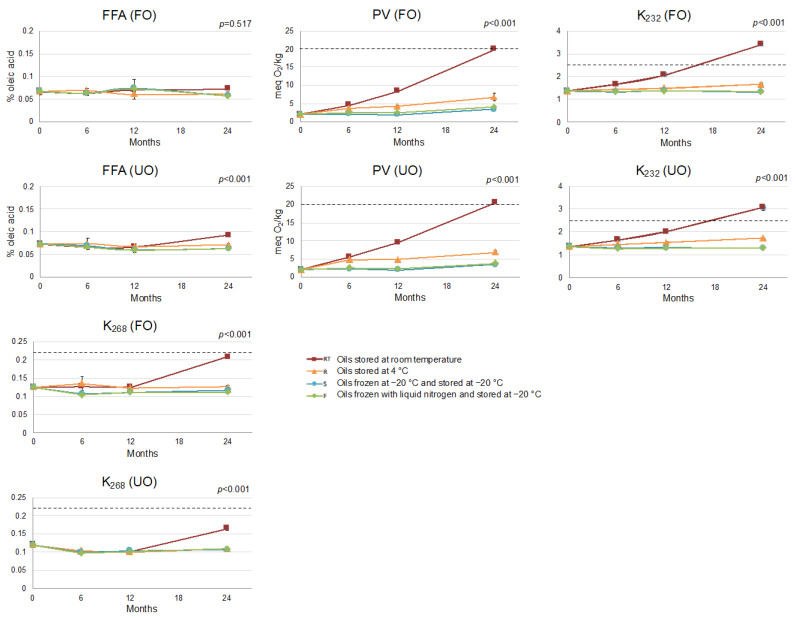
Interaction plot between storage conditions and time for % free fatty acids (FFA), peroxide value (PV), K_232_ and K_268_ of the filtered (FO) and the unfiltered (UO) oils. *p* ≤ 0.05 indicates significant interaction between the two factors (storage conditions and storage time). Error bars correspond to the standard deviation. The established limit for the EVOO category according to EC Regulations is marked by the dotted line.

Figure 1 displays the change in the trade quality indices of the oils over time. The storage conditions had a significantly different impact on the quality parameters of both oils over time (revealed by the significant interactions between the storage conditions and storage time in the two-way ANOVAs on UO and on FO oils separately), except for FFA in FO (Figure 1). In the FO, FFA remained unchanged over the 24-month period at all storage conditions, while in the UO, an increase in FFA was observed at RT after 12 months of storage. This increase could be attributed to the higher water content in the UO (Table 1), which accelerates TAG hydrolysis, as shown in previous studies [24,51].

The values of the oxidative parameters (PV, K_232_ and K_268_) of UO and FO were maintained in the frozen oils (S and F) and clearly increased in the oils stored at RT (Figure 1). This behavior was similar for UO and FO in the case of PV, as the multifactor ANOVA did not reveal a significant interaction between the three factors (filtering × storage conditions × storage time; *p* = 0.526, Supplementary Materials Table S2). However, the interaction of these three factors was significant for K_232_ and K_268_ (*p* = 0.001 and *p* = 0.006, respectively; Supplementary Materials Table S3). Regarding K_232_, the values at RT exceeded the established upper limit for EVOO (according to the European legislation [41]) between 12 and 24 months of storage in both oils, but the values reached at 24 months were higher for FO. The evolution of K_232_ was similar for both types of freezing (S and F). Regarding K_268_, its values also slightly increased at RT between 12 and 24 months both for FO and UO, with the values reached by FO being higher, indicating a greater degree of oxidation. This agreed with the initial higher OSI value in the UO (25.3 h for UO vs. 23.3 h for FO) (Table 1), consistent with previous findings reporting slightly higher oxidative degradation in FO compared to UO [1,12,13]. However, the significant interaction observed for OSI values between filtering × storage conditions × storage time (*p* < 0.001; Supplementary Materials Table S4) revealed a greater decrease in OSI values in UO than in FO when oils were stored at RT and 4 °C, while storage at −20 °C (S and F) maintained the initial values for both oils. To understand this greater OSI decrease in UO, other factors influencing oxidative stability, such as antioxidant content, must be evaluated. Lastly, overall, there was no noticeable difference between the S and F treatments for any of the above-mentioned parameters in either FO or UO (Figure 1).

### 3.2. Phenolic Compounds

The initial values of α-tocopherol and SEC were higher in FO than in UO (Table 1). These results differ from previous studies reporting that filtration does not affect tocopherol content and significantly reduces the total polar phenolic compounds content [2]. Although the effect of filtration may depend on the filtration system [52], the more likely explanation for the results is a rapid deterioration of these compounds in the UO in the few days between production and analysis (the oils were sampled 7 days after production). A concentration effect due to the removal of water may also contribute to the higher concentration in FO of the most lipophilic compounds, such as tocopherols and volatile compounds, but to a much lesser extent.

**Figure 2:**
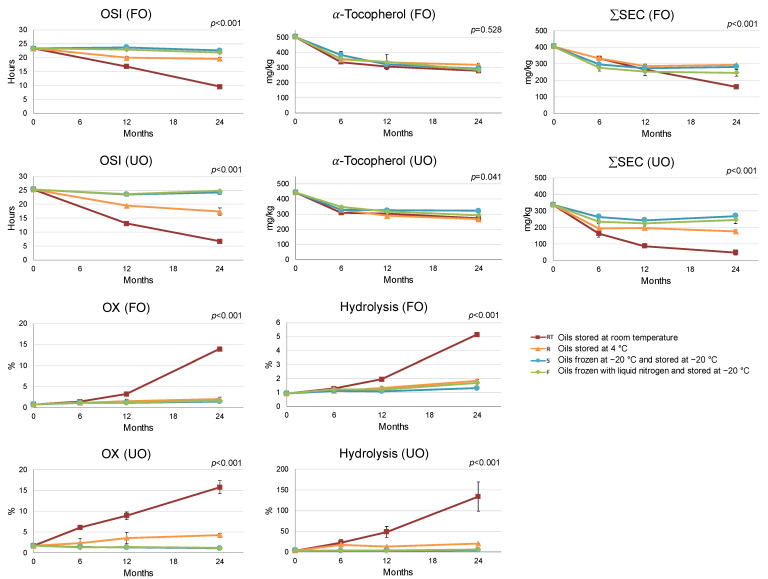
Interaction plot between storage conditions and time for the oxidative stability index (OSI), α-tocopherol content, secoiridoid derivatives content (ΣSEC), oxidation rate of SEC (OX), and hydrolysis rate of SEC (Hydrolysis) of the filtered (FO) and the unfiltered (UO) oils. *p* ≤ 0.05 indicates significant interaction between the two factors (storage conditions and storage time). Error bars correspond to the standard deviation.

The content of α-tocopherol decreased during storage, especially during the first 6 months of storage (Figure 2), without a clear difference between the storage conditions (*p* = 0.528 for FO and *p* = 0.041 for UO). This behavior of α-tocopherol was similar for FO and UO (*p* = 0.089, Supplementary Materials Table S2), confirming the findings of a prior study by Fregapane et al. [12].

Contrarily to the α-tocopherol content, the preservation of SEC compounds largely depended on the storage temperature (Figure 2), in agreement with Díez-Betriu et al. [37]. SEC were better preserved at lower storage temperatures than at RT, but their evolution differed between UO and FO (*p* < 0.001, Supplementary Materials Table S2). Refrigeration temperature was sufficient to maintain the total SEC amount in FO at acceptable levels (27.7% SEC decrease after 24 months), but the same conditions in UO resulted in a 48.1% decrease. Subzero temperatures of −20 °C (S and F oils) preserved SEC in both oils (20.3% and 27.2% SEC decrease after 24 months in FO and UO, respectively). The decrease in SEC observed in UO stored without freezing was primarily caused by the loss of OL derivatives (Supplementary Materials Figure S2). This would justify the greater decrease in OSI of UO during non-frozen storage (Figure 2).

The decrease of SEC in oil can be attributed to both oxidative and hydrolytic degradation through chemical and enzymatic mechanisms. On the one hand, while the presence of water does not have a clear influence on the rate of chemical oxidation reactions, hydrolytic reactions are favored at the oil/water interface [24]. On the other hand, the solids and water in UO can provide a suitable environment for yeasts with phenoloxydase and esterase activity, which are normally present in freshly produced olive oils [53], in order to survive in the UO [1]. This may result in a reduction in polar phenolic compounds, since the activity of phenoloxydases leads to the formation of oxidized forms of *o*-diphenols, and esterases hydrolyze the ester bound of SEC, increasing the content HTy and Ty [53].

Regarding oxidative degradation, Figure 2 displays the changes in the oxidation rate of SEC (OX, measured as the percentage of oxidized forms on the total SEC content, including the oxidized forms). Results show a distinct impact of the storage conditions on FO and UO (*p* < 0.001, Supplementary Materials Table S2); meanwhile, the oxidation rate of SEC in FO was maintained at 4 °C and −20 °C, and in UO it only remained stable at −20 °C, with the values obtained for R and RT being greater.

However, the major impact on SEC content in UO stored without freezing (at R or RT) was due to hydrolytic degradation. The rate of SEC hydrolysis, measured as the percentage of Ty and HTy on the total SEC content, after 24 months was 10.9 (R) and 26 (RT) times higher in UO. These results support previous findings that oil filtration reduces the SEC hydrolysis rate [21]. Conversely, frozen storage effectively maintained the SEC hydrolysis rate so that it was almost unchanged over 24 months in both FO and UO (Figure 2).

In summary, the storage of UO without freezing caused a significant decrease in the SEC content. The main factor for this decrease was the hydrolysis of SEC. However, storing UO at subzero temperatures minimized the negative impact of elevated of water content and microbial activity on the degradation of SEC, effectively extending its shelf life to the same level as FO. The rate at which the oils were frozen (S or F oils) was not found to have any impact on the oxidative or hydrolytic degradation of oils stored at subzero temperatures.

### 3.3. Volatile Compounds

At the beginning of the study, LOX compounds, which are responsible for the desired aroma of olive oils [54], represented 70.8% and 79.1% of the total volatile fraction of the UO and FO, respectively (Table 1). The effect of storage temperature and time on the LOX compounds differed greatly in the two oils (*p* ≤ 0.05 in all cases, Supplementary Materials Table S2).

**Figure 3:**
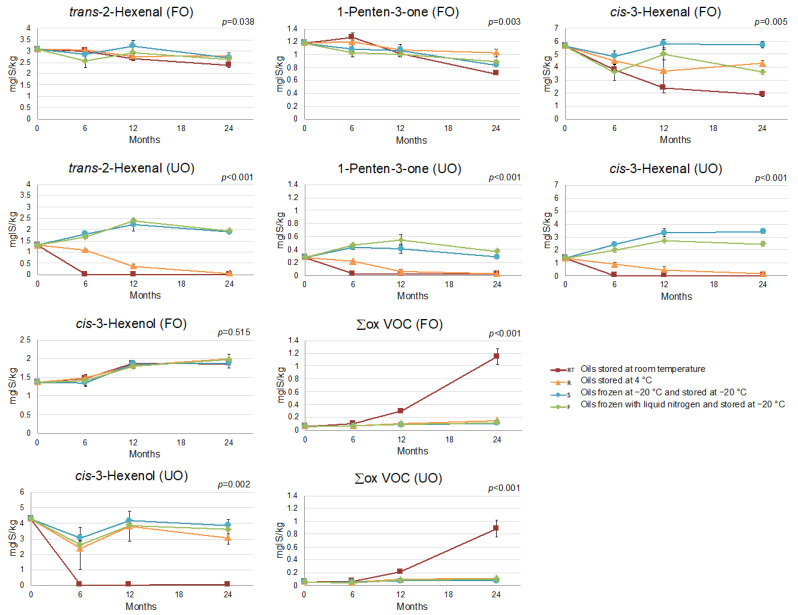
Interaction plot between storage conditions and time for *trans*-2-hexenal, 1-penten-3-one, *cis*-3-hexenal, *cis*-3-hexenol content and the sum of volatile oxidation products (Σox VOC: octane, 1-octene, heptanal, compound with *m/z* 81, 95, 109, 124, according to Vichi et al. [56], octanal, 2-heptenal, nonanal, 2,4-heptadienal) of the filtered (FO) and the unfiltered (UO) oils. *p* ≤ 0.05 indicates significant interaction between the two factors (storage conditions and storage time). Error bars correspond to the standard deviation.

The evolution of LOX compounds in FO during storage agreed with that reported by Díez-Betriu et al. [37], under the same conditions (Figure 3 and Supplementary Materials Figure S3). Briefly, most LOX compounds were not affected by the storage conditions, while the content of compounds deemed to be the major contributors of the desired aroma of olive oil [18,54,55], such as *cis*-3-hexenal and 1-penten-3-one (Figure 3), was reduced with the increment of the storage temperature.

In the case of the UO, an effect of storage temperature was observed in all LOX compounds (Figure 3) except for the pentene dimers (Supplementary Materials Figure S3). At RT, all these compounds were lost after 6 months of storage, while at −20 °C (S and F), their content was either maintained or slightly augmented. Regarding storage at 4 °C, some volatiles (*trans*-2-pentenol, *cis*-2-pentenol, 1-penten-3-one, *trans*-2-hexenal, *trans*-2-hexenol, *cis*-3-hexenal) progressively decreased to 0 at the end of the study, whereas others (1-penten-3-ol, hexyl acetate, *cis*-3-hexenyl acetate, 1-hexanol, *cis*-3-hexenol) were either maintained or slightly augmented. In summary, storage of UO at −20 °C maintained the initial content of all the LOX compounds, and it would be especially indicated to avoid the loss of the LOX compounds that are more susceptible to undergoing deterioration reactions both at 4 °C and RT (*trans*-2-pentenol, *cis*-2-pentenol, 1-penten-3-one, *trans*-2-hexenal, *trans*-2-hexenol, *cis*-3-hexenal) (Figure 3 and Supplementary Materials Figure S3).

Regarding the volatile oxidation products (Σox VOC, Figure 3), the same effect of storage temperature and time was observed in the two oils (*p* = 0.064 of the interaction between filtering × storage conditions × storage time, Supplementary Materials Table S2). In both FO and UO, there was a slight increase over the 24 months of storage at 4 °C and −20 °C, while, at RT, the volatile oxidation products progressively accumulated to a much greater extent.

Lastly, a considerable and unexpected increment in the content of 6-methyl-5-hepten-2-one was detected in both oils (FO and UO) frozen with liquid nitrogen (F) (Supplementary Materials Figure S3). This volatile compound has been associated with fruity, apple and grass aromas [55,57], and an increase in its content has been reported in other conservation studies at RT and refrigeration temperature [37,58,59]. This increment was also detected in the present study at RT and R storage, but it was greater in the F condition, which agrees with a previous study assessing the same storage conditions [37]. These findings suggest that fast-freezing (F) does not entail any improvement over slow-freezing (S) in terms of preserving volatile compounds, and may even alter the volatile fraction of oils stored at subzero temperatures.

### 3.4. Sensory Assessment

**Figure 4:**
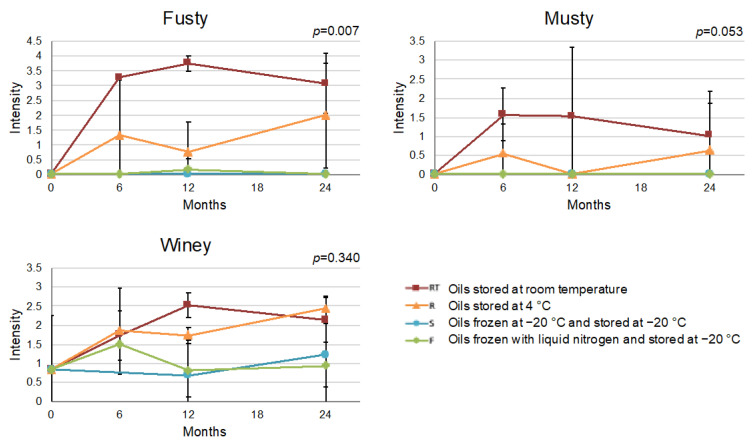
Interaction plot between storage conditions and time for the sensory defects fusty, musty and winey of the unfiltered oil (UO). *p* ≤ 0.05 indicates significant interaction between the two factors (storage conditions and storage time). Error bars correspond to the standard deviation.

Contrarily to the FO, which did not present any sensory defect throughout the storage, the UO presented a median of the winey defect of 0.8 at the beginning of the study, which increased over time at RT and 4 °C (Figure 4). The winey defect is associated with the growth of yeasts, which can be present in recently produced UO [60]. In accordance with Guerrini et al. [19], who described a rapid emergence of fermentative sensory defects even at the very early stages of UO storage, this negative attribute probably developed during the few days (7 days) between production and sampling of the UO. Moreover, during storage at RT and, to a lesser extent, at 4 °C, the musty and fusty defects developed and increased in intensity over time, indicating that under these conditions microbial activity had taken place (Figure 4). On the contrary, during storage at −20 °C, the intensity of the winey defect did not increase and musty and fusty were not detected.

**Figure 5:**
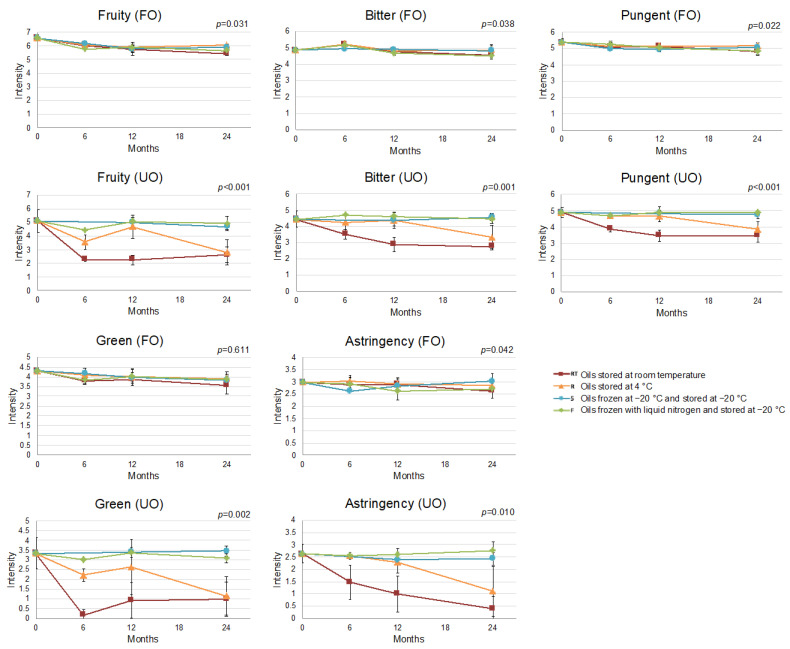
Interaction plot between storage conditions and time for the positive sensory attributes fruity, bitter, pungent, green and astringency of the filtered (FO) and the unfiltered (UO) oils over storage under different conditions. *p* ≤ 0.05 indicates significant interaction between the two factors (storage conditions and storage time). Error bars correspond to the standard deviation.

Regarding the positive sensory attributes, once more, the effect of storage temperature and time depended on the type of oil (*p* ≤ 0.05 in all cases, Supplementary Materials Table S2). In the case of the FO, the positive sensory attributes green, bitter, pungent and astringent either were maintained or slightly decreased, while the fruity attribute experienced a loss from 6.6 to 5.4 at RT but was maintained under cold storage (both under frozen and refrigerated storage) (Figure 5). In contrast, a drastic difference between frozen and non-frozen storage was observed in all the positive attributes of UO. The greatest loss of intensity took place at RT and more gradually at 4 °C, while it was preserved at −20 °C. The decrease in the green and fruity attributes could be ascribed to the reported loss of the LOX compounds (Figure 3), manifesting the relevant contribution of these compounds to the desired aroma of EVOO. Respecting the attributes bitter, astringent and pungent, their loss of intensity at non-freezing temperatures could be related to the observed decrease in some polar phenolic compounds (Figure 2). In fact, the astringent attribute has been linked to Ty and the majority of SEC, while the LIG aglycone content has been associated with the pungent attribute [6]. On the other hand, a positive correlation between OL aglycone and the intensity of the bitter sensation has been reported [61,62]. The results of the sensory assessment of FO and UO at RT are in accordance with the study of Rotondi et al. [20], which evaluated the effect of filtration on 52 VOO during 12 months of storage at RT and concluded that the FO showed a tendency to maintain the positive attributes while they decreased in the UO.

Considering that storage at −20 °C maintained the intensity of these attributes, freezing would be especially recommended for UO, while storage at 4 °C would be insufficient. Freezing with liquid nitrogen would not entail an improvement compared to freezing at −20 °C.

**Figure 6:**
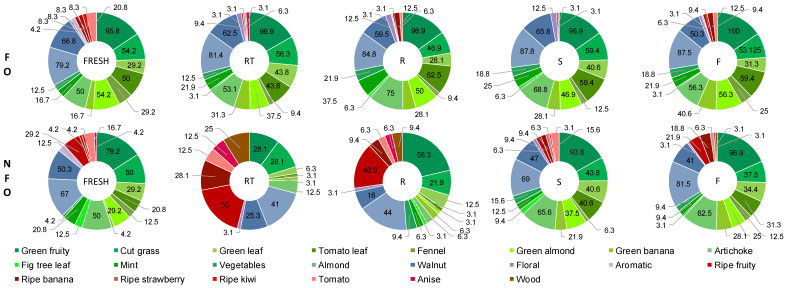
Full profile of secondary sensory attributes of the filtered (FO) and unfiltered (UO) oils at the beginning of the study (fresh) and after 24 months at room temperature (RT), 4 °C (R), and at −20 °C (S, frozen at −20 °C; F, frozen with liquid nitrogen). Secondary sensory attributes are expressed as % of panelists perceiving the attribute. The green and the red color palette correspond to attributes associated with green and ripe notes, respectively.

Since consumer interest towards UO is mainly driven by the particular sensory traits of this product, changes in the full profiles of secondary sensory attributes of the oils were also assessed. Figure 6 shows the secondary sensory profile at time 0 and 24 months at RT, R, S and F. The initial profiles of FO and UO were very similar: both were mainly defined by the attributes green fruity, almond, walnut and cut grass, although they were perceived more intensely (% of panelists) in the FO. Moreover, both were principally described by attributes related to green notes (10 and 11 green attributes in FO and UO, respectively). The main difference between the initial profiles of the two oils was the higher % of panelists perceiving the ripe fruity attribute in the UO.

The secondary sensory attributes profile of the FO remained practically unchanged after 24 months at the three storage temperatures and the two freezing speeds (Figure 6), coinciding with the results observed for the main positive sensory attributes. Only a slight decrease in the % of panelists perceiving the tomato leaf note and a slight increase in the % of panelists perceiving the anise note were observed in the RT oil after 24 months of storage.

On the other hand, a strong effect of storage temperature on the sensory profile of UO was observed (Figure 6). Firstly, the % of panelists perceiving some of the main initial attributes dramatically decreased as the storage temperature increased. In particular, storage temperature negatively affected the attributes associated with green notes, which are depicted in the figure using a green palette; the number of green notes and the % of panelists perceiving them decreased as the storage temperature increased. This was accompanied by an increase in the % of panelists perceiving attributes associated with ripe notes (depicted in the figure with a red color palette), mainly ripe fruity and ripe banana. Lastly, the wood attribute, which was not detected initially, increased with the storage temperature. Nevertheless, UO stored at −20 °C (both S and F) maintained the initial sensory profile in terms of complexity (number of green notes) and intensity (% of panelists perceiving the attributes). Thus, by storing the UO at −20 °C, its characteristic sensory profile was preserved, which would drastically change at non-freezing temperatures. Regarding the speed of freezing, a slightly higher % of panelists perceived ripe notes in the F oil compared to the S oil, reflecting no advantages of the fast-freezing over the slow-freezing.

### 3.5. Effect of Storing at Room Temperature after Thawing

Some authors suggested that the physical state changing of the EVOO resulting from temperature variation could affect its resistance to oxidation processes, which could lead to a faster loss of quality after thawing [30,36]. For this reason, the impact of freezing on the quality of FO and UO after thawing and storing at RT was evaluated. Because slow-freezing was established as the most suitable freezing method, the stability of oils frozen using this method and then thawed was compared to that of freshly produced oils. The focus of the comparison was on physicochemical quality indices and sensory attributes, which did not undergo substantial changes during the frozen storage period. The effect of thawing (TH) and storage time (T) on the loss of quality of the oils was evaluated by the interaction between these factors (TH*T) assessed by two-way ANOVA (Table 2). The effect of single factors (TH, T) is reported in Supplementary Materials (Tables S3 and S4).

The effect of thawing on the stability of FO and UO during RT storage was not significant according to most of the physicochemical indices (Table 2). The significant effect observed for PV and K_268_ in FO and for FFA in UO corresponds to very small variations in the values of these parameters that could be considered as negligible. Interestingly, sensory attributes such as fruity, pungent, green, astringency, and the overall score were better preserved after 6 months of RT storage if UO had been previously frozen at −20 °C and then thawed. This effect is less noticeable in the case of FO. This could be attributed to the decrease in the viability of microorganisms and to a lower enzymatic activity in the UO following a year-long freezing period, resulting in reduced microbial degradation of the oil during post-thaw preservation. While this phenomenon impacts sensory attributes that are highly susceptible to the presence of microorganisms, it has no effect on physicochemical oxidation indices, reinforcing the hypothesis that the positive impact of frozen storage and thawing on UO stability is related to the reduction in microbiological activity.

Although the impact of frozen storage on reducing microbiological or enzymatic spoilage in thawed UO remains to be confirmed through further research, current results indicate that contrary to previous studies [30,36], the freezing and thawing process did not have a negative impact on the stability of UO during subsequent storage.

## 4. Conclusions

The results of the present study confirmed the low stability of UO over conventional storage at RT: while secondary oxidation products (K_268_ and volatile oxidation products) were slightly lower in UO than in FO, its higher water content led to an increase in FFA and a decrease in SEC, OSI and the bitter sensory attribute. Likewise, LOX volatiles drastically decreased over time, causing a severe decrease in the intensity of fruity and green attributes, as well as a strong shift from green to ripe notes in the secondary sensory profile. Lastly, while no sensory defects were detected in the FO, the winey, musty and fusty defects increased during the first 12 months in the UO, probably due to the presence of yeasts. As expected, cold storage preserved both oils better than conventional RT storage, as observed for trade quality parameters, polar phenols, OSI, volatile compounds and sensory attributes. However, while refrigeration temperature was sufficient to maintain almost unaltered characteristics of FO during storage, at non-freezing temperature, UO experienced a severe decrease in its sensory quality compared to FO, mainly due to hydrolysis of SEC and degradation of the volatile fraction. Only storage at −20 °C allowed to effectively preserve UO quality traits. These results indicate that degradation phenomena related to the higher water content and microbial activity of UO can be minimized with frozen storage, which proved to be a suitable strategy to increase the shelf-life of UO. Fast-freezing did not improve the preservation of UO compared to slow-freezing and even induced alterations in the volatile fraction of oils stored at subzero temperatures. Finally, the freezing and thawing process did not have a negative impact on the stability of UO during subsequent storage. Rather, frozen storage may potentially reduce microbiological and enzymatic spoilage in thawed UO, but this needs to be confirmed through additional research.

## Figures and Tables

**Table 1:** Selected parameters of the initial characterization of the filtered (FO) and unfiltered (UO) oils in the study. Values correspond to mean values of two experimental replicates (all parameters were further determined in analytical duplicate, except for K_232_, K_268_ and ∆K which were determined in triplicate). The full initial characterization of the oils can be found in Supplementary Materials, Table S1.

	FO	UO
FFA%	0.067	0.073
PV (meq O_2_/kg)	2.0	2.1
K_232_	1.37	1.35
K_268_	0.13	0.12
Moisture and volatile matter (%)	0.036	0.376
OSI (h)	23.3	25.3
**Polar and lipophilic phenolic compounds** **(mg/kg)**		
α-Tocopherol	504	444.5
Hydroxytyrosol (3,4-DHPEA)	1.17	7.24
Tyrosol (*p*-HPEA)	2.61	4.67
ΣSEC	405.2	337.9
ΣOL	248.6	188.6
ΣLIG	156.6	149.3
Σox SEC	3.02	5.88
**Volatile compounds (mg IS/kg oil)**		
ΣLOX VOC	16.1	12.7
Σox VOC	0.11	0.2
Total volatile compounds	17.9	16.2
**Sensory analysis** ^1^		
Fruity	6.6	5.1
Bitter	4.9	4.4
Pungent	5.4	4.9
Green	4.3	3.3
Astringency	3.0	2.6

^1^ Median of intensity (0–10 scale). Abbreviations: FFA, free fatty acids; PV, peroxide value; OSI, oxidative stability index; ΣSEC, sum of secoiridoid derivatives; ΣOL, sum of oleuropein derivatives; ΣLIG, sum of ligstroside derivatives; Σox SEC, sum of oxidized forms of secoiridoid derivatives; IS, internal standard; ΣLOX VOC, sum of volatile compounds from lipoxygenase pathway; Σox VOC, sum of volatile oxidation products (octane, 1-octene, pentanal, heptanal, 1-pentanol, octanal, 2-heptenal, nonanal, 2,4-heptadienal).

**Table 2:** Changes in physicochemical and sensory quality parameters (mean values) assessed in fresh (control) and thawed filtered (FO) and unfiltered (UO) oils after 6 months of storage at room temperature and the effect of the interaction (*p* values) between thawing and storage time (TH*T) assessed by two-way ANOVA.

	FO					UO				
	CT_0_^1^	CT_6_^1^	THT_0_^1^	THT_6_^1^	TH*T	CT_0_^1^	CT_6_^1^	THT_0_^1^	THT_6_^1^	TH*T
FFA (% oleic acid)	0.07	0.06	0.07	0.06	0.830	0.07	0.07	0.06	0.08	0.027
PV (meq O_2_/kg)	2.0	4.5	1.8	4.8	0.031	2.1	5.5	2.0	5.3	0.389
K_232_	1.37	1.65	1.38	1.65	0.727	1.35	1.64	1.32	1.57	0.546
K_268_	0.13	0.13	0.11	0.13	0.005	0.12	0.10	0.10	0.10	0.479
**Positive sensory attributes ^2^**										
Fruity	6.6	6.0	5.8	5.9	0.016	5.1	2.3	5.0	4.0	0.001
Bitter	4.9	5.2	4.9	4.9	0.109	4.4	3.6	4.4	4.1	0.203
Pungent	5.4	5.1	4.9	5.1	0.003	4.9	3.9	4.8	4.4	0.019
Green	4.3	3.8	4.0	3.8	0.103	3.3	0.1	3.4	2.7	<0.001
Astringency	3.0	2.9	2.8	3.0	0.204	2.6	1.3	2.4	2.1	0.150
**Global sensory score ^3^**	7.8	7.5	7.5	7.6	0.007	7.2	4.9	7.2	6.4	<0.001

^1^ Results from two experimental replicates (all parameters were further determined in analytical duplicate, except for K_232_ and K_268_ which were determined in triplicate). ^2^ Median of intensity (0–10 scale). ^3^ 0–9 scale, estimated by means of an algorithm based on the panels’ outputs, according to Romero [63]. Abbreviations: CT_x_, control oil stored at room temperature; THT_x_, oil frozen at −20 °C, stored at −20 °C, thawed and stored at room temperature; T_0_: 0 months of storage at room temperature; T_6_: 6 months of storage at room temperature; TH, thawing; T, storage time; FFA, free fatty acids; PV, peroxide value.

## Data Availability

The datasets generated for this study are available on request to the corresponding author.

## Supplementary Materials

The following supporting information can be downloaded at https://www.mdpi.com/article/10.3390/foods12061228/s1, Table S1: Initial characterization (quality indices. fatty acid profile, moisture, oxidative stability index, tocopherols, polar phenolic compounds, volatile compounds and sensory analysis) of the filtered (FO) and unfiltered (UO) oils in the study. Values correspond to mean values of two experimental replicates (all parameters were further determined in analytical duplicate, except for K_232_, K_268_ and ∆K that were determined in triplicate); Table S2. *p*-Values of the multifactor ANOVA assessing the effect of filtration (FI), storage conditions (SC), and storage time (T) and their interactions on the trade quality parameters, oxidative stability index (OSI), phenolic compounds, volatile compounds and sensory assessment of the FO and the UO (*n* = 64); Table S3: Changes in the physicochemical and sensory quality parameters (mean values) assessed in fresh (control) and thawed filtered oil (FO) after 6 months of storage at room temperature. Effect of thawing (TH), storage time (T) and the interaction between them (TH*T) (p values) assessed by two-way ANOVA; Table S4: Changes in physicochemical and sensory quality parameters (mean values) assessed in fresh (control) and thawed unfiltered oil (UO) after 6 months of storage at room temperature. Effect of thawing (TH), storage time (T) and the interaction between them (TH*T) (*p* values) assessed by two-way ANOVA; Figure S1. Scheme of the experimental design of (a) the conservation study of filtered oil (FO) and unfiltered oil (UO); (b) the study to evaluate the effect of freezing and thawing on the stability of FO and UO; Figure S2. Interaction plot between storage conditions and time for the oleuropein and ligstroside derivatives content (ΣOL and ΣLIG, respectively) of the filtered (FO) and the unfiltered (UO) oils. *p* ≤ 0.05 indicates significant interaction between the two factors (storage conditions and storage time). Error bars correspond to the standard deviation; Figure S3. Interaction plot between storage conditions and time for *cis*-2-pentenol, *trans*-2-pentenol, 1-penten-3ol, 1-hexanol, *trans*-2-hexenol, hexyl acetate, *cis*-3-hexenyl acetate and 6-methyl-5-hepten-2-one content of the filtered (FO) and the unfiltered (UO) oils. *p* ≤ 0.05 indicates significant interaction between the two factors (storage conditions and storage time). Error bars correspond to the standard deviation.
