# Supplementary material for: Subzero Temperature Storage to Preserve the Quality Attributes of Veiled Virgin Olive Oil
Source: Foods. 2023 Mar 13;12(6):1228. doi: 10.3390/foods12061228 (PMC10048678; doi:10.3390/foods12061228)

Supplementary Materials

Subzero Temperature Storage to Preserve the Quality Attributes of Veiled Virgin Olive Oil

Anna Díez-Betriu [1,2], Agustí Romero [3], Antonia Ninot [3], Alba Tres [1,2], Stefania Vichi [1,2,*] and Francesc Guardiola [1,2]

[1] Departament de Nutrició, Ciències de l'Alimentació i Gastronomia, Campus de l'Alimentació de Torribera, Facultat de Farmàcia i Ciències de l'Alimentació, Universitat de Barcelona,
08921 Santa Coloma de Gramenet, Spain; annadiez@ub.edu (A.D.-B.); atres@ub.edu (A.T.); stefaniavichi@ub.edu; fguardiola@ub.edu (F.G.)
[2] Institut de Recerca en Nutrició i Seguretat Alimentària (INSA-UB), Universitat de Barcelona (UB),
08921 Santa Coloma de Gramenet, Spain
[3] Institute of Agrifood Research and Technology (IRTA), Mas Bové Ctra. Reus-El Morell km 3.8,
43120 Constantí, Spain; agusti.romero@irta.cat (A.R.); antonia.ninot@irta.cat (A.N.)
* Correspondence: stefaniavichi@ub.edu

Table S1. Initial characterization (quality indices, fatty acid profile, moisture, oxidative stability index, tocopherols, polar phenolic compounds, volatile compounds and sensory analysis) of the filtered (FO) and unfiltered (UO) oils in the study. Values correspond to mean values of two experimental replicates (all parameters were further determined in analytical duplicate, except for K_{232}, K_{268} and ΔK that were determined in triplicate).

	FO	UO
FFA%	0.067	0.073
PV (meq O_2/kg)	2.0	2.1
K_{232}	1.37	1.35
K_{268}	0.13	0.12
ΔK	0.008	0.007
Md	0.0	0.8
Mf	6.6	5.1
Palmitic acid (C16:0) (%)	13.3	13.4
Oleic acid (C18:1 n-9) (%)	77.1	76.9
Linoleic acid (C18:2 n-6) (%)	3.81	3.79
Linolenic acid (C18:3 n-3) (%)	0.76	0.76
SFA (%)	16.7	16.9
Oleic/linoleic ratio	20.2	20.3
Moisture and volatile matter (%)	0.036	0.376
OSI (h)	23.3	25.3
**Polar and lipophilic phenolic compounds (mg/kg)**		
α-Tocopherol	504.0	444.5

	FO	UO
β-Tocopherol	3.32	2.95
γ-Tocopherol	10.6	9.5
ΣPolar phenolic compounds	426.3	370.8
Hydroxytyrosol (3,4-DHPEA)	1.17	7.24
Tyrosol (*p*-HPEA)	2.61	4.67
3,4-DHPEA-EDA, oxidized form	0.04	0.04
3,4-DHPEA-EDA	56.5	42.4
3,4-DHPEA-EDA, oxidized form	0.04	0.09
Oleuropein	57.2	37.8
3,4-DHPEA-EA, dialdehyde form	69.0	44.3
Tyrosyl acetate	6.29	5.10
p-HPEA-EDA, oxidized form	0.00	0.00
p-HPEA-EDA	70.2	65.7
Pinoresinol + 1-Acetoxypinoresinol	2.53	3.03
p-HPEA-EDA, oxidized form	0.00	0.00
3,4-HPEA-EA, oxidized aldehyde and hydroxylic form	0.42	0.41
3,4-HPEA-EA, aldehyde and hydroxylic form	65.9	64.1
p-HPEA-EA, oxidized aldehyde and hydroxylic form	2.52	5.34
p-HPEA-EDA, aldehyde and hydroxylic form	86.4	83.7
Luteolin	3.68	5.16
Apigenin	1.75	1.82
EFSA CLAIM	409.0	349.8
ΣSEC	405.2	337.9
ΣOL	248.6	188.6
ΣLIG	156.6	149.3
Σox SEC	3.02	5.88
**<u>Volatile compounds</u> (mg IS/kg oil)**		
**LOX C_5 compounds**		
PD1	0.18	0.18
PD2	0.10	0.10
PD3	0.71	0.70
PD4	0.49	0.54
PD5	0.16	0.17
PD6	0.26	0.27
PD7	0.24	0.29
1-penten-3-ol	0.30	0.23
trans-2-pentenol	0.09	0.05
cis-2-pentenol	0.53	0.40
trans-2-pentenal	0.21	0.09

	FO	UO
1-penten-3-one	1.18	0.28
**LOX C_6 aldehydes**		
Hexanal	0.57	0.36
cis-3-Hexenal	5.64	1.37
trans-2-Hexenal	3.07	1.31
**LOX C_6 alcohols**		
1-Hexanol	0.30	0.88
trans-3-Hexenol	0.05	0.21
cis-3-Hexenol	1.37	4.30
trans-2-Hexenol	0.03	0.27
**LOX C_6 esters**		
Hexyl acetate	0.05	0.06
cis-3-Hexenyl acetate	0.56	0.61
**Oxidation products**		
Octane	0.04	0.04
1-Octene	0.00	0.00
Pentanal	0.03	0.02
Heptanal	0.00	0.00
1-Pentanol	0.01	0.11
Octanal	0.00	0.00
2-Heptenal	0.00	0.00
Nonanal	0.01	0.01
2,4-Heptadienal	0.00	0.00
**Others**		
Methyl acetate	0.05	0.22
Ethyl acetate	0.11	1.59
2-Butanone	0.01	0.00
2-Methylbutanal	0.00	0.00
3-Methylbutanal	0.00	0.00
1-Methoxyhexane	0.13	0.12
3-Pentanol	0.01	0.02
3-Pentanone	0.17	0.56
trans-4,8-Dimethyl-1,3,7-nonatriene	0.02	0.02
6-Metil-5-hepten-2-one	0.00	0.00
cis,trans-2,4-Hexadienal	0.37	0.22
trans,trans-2,4-Hexadienal	0.79	0.53
2-ethyl Hexanol	0.01	0.01
α-Copaene	0.01	0.01
Benzaldehyde	0.02	0.02
**ΣPD**	2.14	2.25
**ΣC_5 alcohols**	0.92	0.69

	FO	UO
**ΣC_5 ketones**	1.18	0.28
**ΣC_6 aldehydes**	9.28	3.04
**ΣC_6 alcohols**	1.75	5.66
**ΣC_6 esters**	0.62	0.67
**ΣLOX compounds**	16.10	12.67
**Σox VOC**	0.11	0.20
**Total volatile compounds**	17.92	16.18
**Sensory analysis**		
**Positive attributes**[1]		
Fruity	6.6	5.1
Bitter	4.9	4.4
Pungent	5.4	4.9
Apple	0.3	0.0
Ripe fruits	0.0	0.0
Green	4.3	3.3
Sweet	3.9	4.1
Astringency	3.0	2.6
Almond	2.7	1.9
Walnut	1.8	0.7
Other positive attributes	3.2	2.6
**Global sensory score**[2]	7.8	7.2
**Complexity**[3]	6.0	4.7
**Secondary sensory attributes**[4]		
Green fruity	95.8	79.2
Ripe fruity	8.3	29.2
Ripe banana	8.3	4.2
Ripe kiwi	8.3	4.2
Cut grass	54.2	50.0
Green leaf	29.2	29.2
Defects	0.0	4.0
Tomato leaf	50.0	20.8
Tomato	20.8	16.7
Almond	79.2	67.0
Walnut	66.8	50.3
Fennel	29.2	12.5
Green almond	54.2	29.2
Banana	16.7	4.2
Artichoke	50.0	50.0

	FO	UO
Floral	4.2	0.0
Aromatic	8.3	12.5
Anise	0.0	4.2
Fig tree leaf	0.0	12.5
Mint	16.7	20.8
Vegetables	12.5	4.2

[1] Median of intensity (0-10 scale); [2] 0-9 scale, estimated by means of an algorithm based on the panels' outputs, according to Romero [1]. [3] Number of secondary sensory attributes perceived by more than 30% of the panelists; [4] Percentage of panelists perceiving the attribute.

Abbreviations: FFA, free fatty acids; PV, peroxide value; Md, Median of defects; Mf, Median of fruity notes; SFA, saturated fatty acids; OSI, oxidative stability index; ND, not detected; 3,4-DHPEA-EDA, dihaldehyde form of decarboxymethyl oleuropein algycone; 3,4-DHPEA-EA, dialdehyde form of oleuropein aglycone; *p*-HPEA-EDA, dihaldehyde form of decarboxymethyl ligstroside algycone; *p*-HPEA-EA, dialdehyde form of ligstroside aglycone; EFSA CLAIM, sum of hydroxytyrosol, tyrosol and derivatives; ΣSEC, sum of secoiridoid derivatives; ΣOL, sum of oleuropein derivatives; ΣLIG, sum of ligstroside derivatives; Σox SEC, sum of oxidized forms of secoiridoid derivatives; IS, internal standard; LOX, lipoxygenase pathway; PD, pentene dimer; Σox VOC, sum of volatile oxidation products (octane, 1-octene , pentanal, heptanal, 1-pentanol, octanal, 2-heptenal, nonanal, 2,4-heptadienal).

Table S2. *p*-Values of the multifactor ANOVA assessing the effect of filtration (FI), storage conditions (SC), and storage time (T) and their interactions on the trade quality parameters, oxidative stability index (OSI), phenolic compounds, volatile compounds and sensory assessment of the FO and the UO (*n*=64).

	FI	SC	T	FI*SC	SC*T	FI*T	FI*SC*T
FFA	0.008	0.018	0.263	0.325	0.002	0.002	0.753
PV	0.001	<0.001	<0.001	0.006	<0.001	0.100	0.526
K_{232}	0.001	<0.001	<0.001	0.001	<0.001	0.102	0.001
K_{268}	<0.001	<0.001	<0.001	<0.001	<0.001	0.002	0.006
OSI	0.046	<0.001	<0.001	<0.001	<0.001	<0.001	<0.001
α-Tocopherol	<0.001	0.006	<0.001	0.104	0.514	<0.001	0.089
ΣSEC	<0.001	<0.001	<0.001	<0.001	<0.001	0.019	<0.001
ΣOL	<0.001	<0.001	<0.001	<0.001	<0.001	0.001	<0.001
ΣLIG	<0.001	<0.001	<0.001	<0.001	<0.001	0.556	0.142
Hydrolysis	<0.001	<0.001	<0.001	<0.001	<0.001	<0.001	<0.001
OX	<0.001	<0.001	<0.001	<0.001	<0.001	0.024	<0.001
**Volatile compounds**							
1-Penten-3-one	<0.001	<0.001	<0.001	<0.001	<0.001	<0.001	<0.001
1-Penten-3-ol	<0.001	<0.001	<0.001	<0.001	0.004	<0.001	<0.001
cis-2-Pentenol	<0.001	<0.001	<0.001	<0.001	<0.001	<0.001	<0.001
trans-2-Pentenol	<0.001	<0.001	<0.001	<0.001	<0.001	<0.001	<0.001
trans-2-Hexenal	<0.001	<0.001	<0.001	<0.001	<0.001	0.382	<0.001
cis-3-Hexenal	<0.001	<0.001	<0.001	0.001	<0.001	<0.001	0.073
1-Hexanol	<0.001	<0.001	<0.001	<0.001	0.010	<0.001	0.008
cis-3-Hexenol	<0.001	<0.001	<0.001	<0.001	<0.001	<0.001	<0.001
trans-2-Hexenol	<0.001	<0.001	<0.001	<0.001	<0.001	<0.001	<0.001
Hexyl acetate	<0.001	<0.001	<0.001	<0.001	<0.001	<0.001	<0.001
cis-3-Hexenyl acetate	<0.001	<0.001	<0.001	<0.001	<0.001	<0.001	<0.001

	FI	SC	T	FI*SC	SC*T	FI*T	FI*SC*T
6-Methyl-3-hepten-2-one	0.016	<0.001	<0.001	0.082	0.001	0.180	0.788
Σox VOC	<0.001	<0.001	<0.001	<0.001	<0.001	0.003	0.064
**Sensory assessment**							
Fruity	<0.001	<0.001	<0.001	<0.001	<0.001	0.012	<0.001
Bitter	<0.001	<0.001	<0.001	<0.001	<0.001	0.007	<0.001
Pungent	<0.001	<0.001	<0.001	<0.001	<0.001	0.130	<0.001
Green	<0.001	<0.001	<0.001	<0.001	<0.001	0.014	0.001
Astringency	<0.001	<0.001	<0.001	<0.001	0.002	0.007	0.007

Abbreviations: FFA, free fatty acids; PV, peroxide value; OSI, oxidative stability index; ΣSEC, sum of secoiridoid derivatives; ΣOL, sum of oleuropein derivatives; ΣLIG, sum of ligstroside derivatives; OX, oxidation rate of secoiridoid derivatives; Σox VOC, sum of volatile oxidation products (heptanal, octane, octanal, 1-octene, 2-heptenal, 2,4-heptadienal, compound not identified 1, nonanal).

Table S3. Changes in physicochemical and sensory quality parameters (mean values) assessed in fresh (control) and thawed filtered (FO) oils after 6 months of storage at room temperature. Effect of thawing (TH), storage time (T) and the interaction between them (TH*T) (*p* values) assessed by two-way ANOVA.

	**FO Control**		**FO Thawed**		**TH**[2]	**T**[2]	**TH*T**[2]
	**CT_0**[1]	**CT_6**[1]	**THT_0**[1]	**THT_6**[1]			
FFA (% oleic acid)	0.07	0.06	0.07	0.06	0.513	0.087	0.830
PV (meq O_2/kg)	2.0	4.5	1.8	4.8	0.358	<0.001	0.031
K_{232}	1.37	1.65	1.38	1.65	0.597	<0.001	0.727
K_{268}	0.13	0.13	0.11	0.13	0.006	0.017	0.005
**Positive sensory attributes**[3]							
Fruity	6.6	6.0	5.8	5.9	0.006	0.065	0.016
Bitter	4.9	5.2	4.9	4.9	0.197	0.128	0.109
Pungent	5.4	5.1	4.9	5.1	0.003	0.147	0.003
Green	4.3	3.8	4.0	3.8	0.071	0.010	0.103
Sweet	3.9	3.8	3.8	3.7	0.311	0.208	0.894
Astringency	3.0	2.9	2.8	3.0	0.749	0.716	0.204
Almond	2.7	2.5	2.3	2.5	0.071	0.916	0.151
**Global sensory score**[4]	7.8	7.5	7.5	7.6	0.021	0.040	0.007
**Complexity**[5]	7.0	8.0	7.5	9.0	0.251	0.089	0.678

[1] Results from two experimental replicates (all parameters were further determined in analytical duplicate, except for K_{232} and K_{268} that were determined in triplicate). [2] *p* values of the main effects (TH, thawing and T, storage at room temperature) and their interaction (TH*T) assessed by two-way ANOVA. [3] Median of intensity (0-10 scale). [4] 0-9 scale, estimated by means of an algorithm based on the panels' outputs, according to Romero [1]. [5] Number of secondary sensory attributes perceived by more than 30% of the panelists. Abbreviations: CT_x, control oil stored at room temperature; THT_x, oil frozen at −20 °C, stored at −20 °C, thawed and stored at room temperature; T_0: 0 months of storage at room temperature; T_6: 6 months of storage at room temperature; TH, thawing; T, storage time; FFA, free fatty acids; PV, peroxide value.

Table S4. Changes in physicochemical and sensory quality parameters (mean values) assessed in fresh (control) and thawed unfiltered (UO) after 6 months of storage at room temperature. Effect of thawing (TH), storage time (T) and the interaction between them (TH*T) (p values) assessed by two-way ANOVA.

	**UO Control**		**UO Thawed**		**TH**[2]	**T**[2]	**TH*T**[2]
	**CT_0**[1]	**CT_6**[1]	**THT_0**[1]	**THT_6**[1]			
FFA (% oleic acid)	0.07	0.07	0.06	0.08	0.777	0.103	0.027
PV (meq O_2/kg)	2.1	5.5	2.0	5.3	0.297	0.042	0.389
K_{232}	1.35	1.64	1.32	1.57	0.160	0.053	0.546
K_{268}	0.12	0.10	0.10	0.10	0.619	0.183	0.479
**Positive sensory attributes**[3]							
Fruity	5.1	2.3	5.0	4.0	0.001	<0.001	0.001
Bitter	4.4	3.6	4.4	4.1	0.280	0.024	0.203
Pungent	4.9	3.9	4.8	4.4	0.037	0.001	0.019
Green	3.3	0.1	3.4	2.7	<0.001	<0.001	<0.001
Sweet	4.1	4.0	4.0	4.1	0.708	0.940	0.474
Astringency	2.6	1.3	2.4	2.1	0.405	0.044	0.150
Almond	1.9	0.0	2.3	0.7	0.065	0.001	0.524
**Global sensory score**[4]	7.2	4.9	7.2	6.4	<0.001	<0.001	<0.001
**Complexity**[5]	5.0	2.5	7.0	5.5	0.015	0.031	0.460

[1] Results from two experimental replicates (all parameters were further determined in analytical duplicate, except for K_{232} and K_{268} that were determined in triplicate). [2] p values of the main effects (TH, thawing and T, storage at room temperature) and their interaction (TH*T) assessed by two-way ANOVA. [3] Median of intensity (0-10 scale). [4] 0-9 scale, estimated by means of an algorithm based on the panels' outputs, according to Romero [1]. [5] Number of secondary sensory attributes perceived by more than 30% of the panelists. Abbreviations: CT_x, control oil stored at room temperature; THT_x, oil frozen at −20 °C, stored at −20 °C, thawed and stored at room temperature; T_0: 0 months of storage at room temperature; T_6: 6 months of storage at room temperature; TH, thawing; T, storage time; FFA, free fatty acids; PV, peroxide value.

Reference

1. Romero, A. Caracterización y diferenciación de los aceites vírgenes de oliva de la comarca del Priorat (Tarragona) dentro del mercado global de aceites de la variedad 'Arbequina'. Tesis doctoral, UdL 2011. Available online: https://tdx.cat/handle/10803/77835#page=70 (accessed on March, 9th 2023).

Figure S1. Scheme of the experimental design of (a) the conservation study of filtered oil (FO) and unfiltered oil (UO); (b) the study to evaluate the effect of freezing and thawing on the stability of FO and UO.

a) Experimental design: conservation study

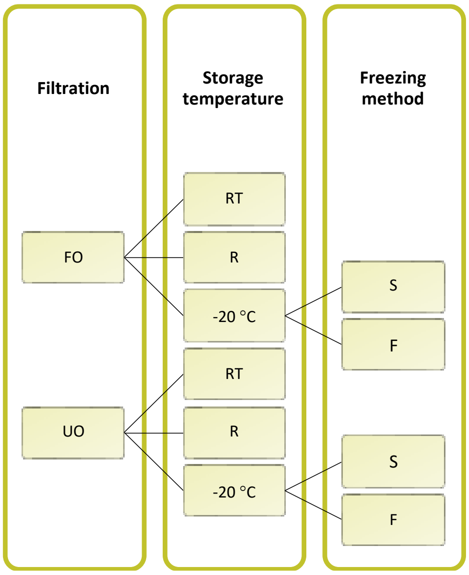

Storage in the dark for 24 months

Parameter	0	6	12	24
Fatty acid composition	✓			
Moisture and volatile matter	✓			
Trade quality parameters	✓	✓	✓	✓
Sensory analysis	✓	✓	✓	✓
Oxidative Stability Index	✓		✓	✓
α-Tocopherol content	✓	✓	✓	✓
Polar phenolic compounds	✓	✓	✓	✓
Volatile compounds	✓	✓	✓	✓

Abbreviations: FO, filtered oil; UO, unfiltered oil; RT, oils stored at room temperature; R, oils stored at 4°C; S, oils frozen at −20°C and stored at −20°C; F, oils frozen with liquid nitrogen and stored at −20°C.

b) Experimental design: effect of freezing and thawing on the stability of FO and UO.

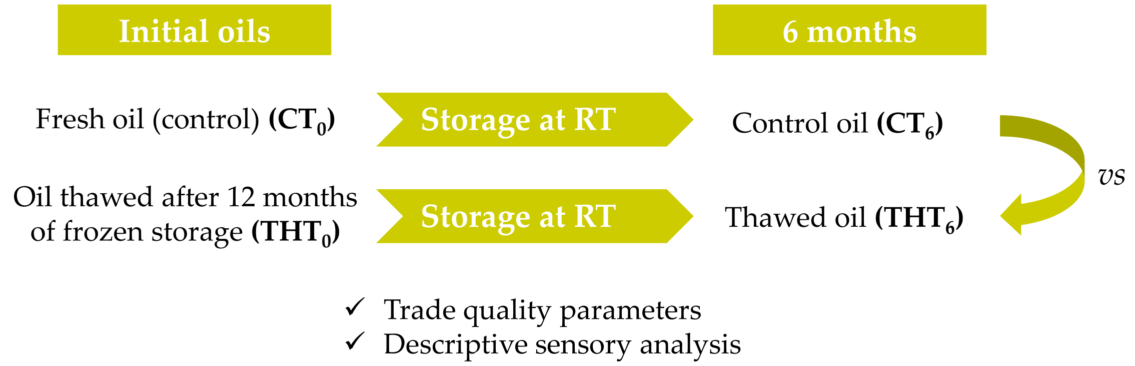

Abbreviations: RT, room temperature; CTx, control oil stored at room temperature; THTx, oil frozen at −20 °C, stored at −20 °C, thawed and stored at room temperature; T_0: 0 months of storage at room temperature; T_6: 6 months of storage at room temperature.

Figure S2. Interaction plot between storage conditions and time for the oleuropein and ligstroside derivatives content (∑OL and ∑LIG, respectively) of the filtered (FO) and the unfiltered (UO) oils. p≤0.05 indicates significant interaction between the two factors (storage conditions and storage time). Error bars correspond to the standard deviation.

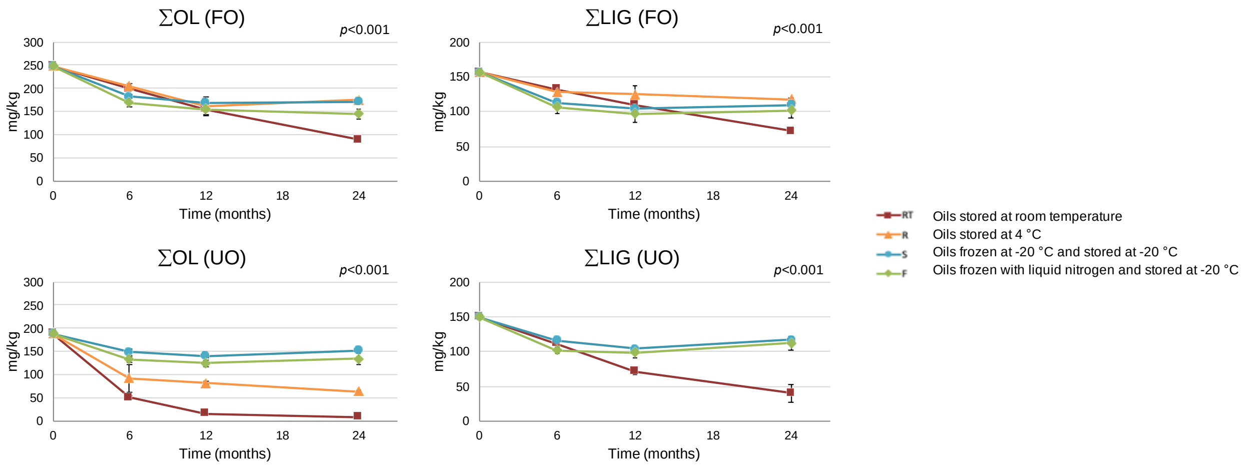

Figure S3. Interaction plot between storage conditions and time for *cis*-2-pentenol, *trans*-2-pentenol, 1-penten-3-ol, 1-hexanol, *trans*-2-hexenol, hexyl acetate, *cis*-3-hexenyl acetate and 6-methyl-5-hepten-2-one content of the filtered (FO) and the unfiltered (UO) oils. $p \leq 0.05$ indicates significant interaction between the two factors (storage conditions and storage time). Error bars correspond to the standard deviation.

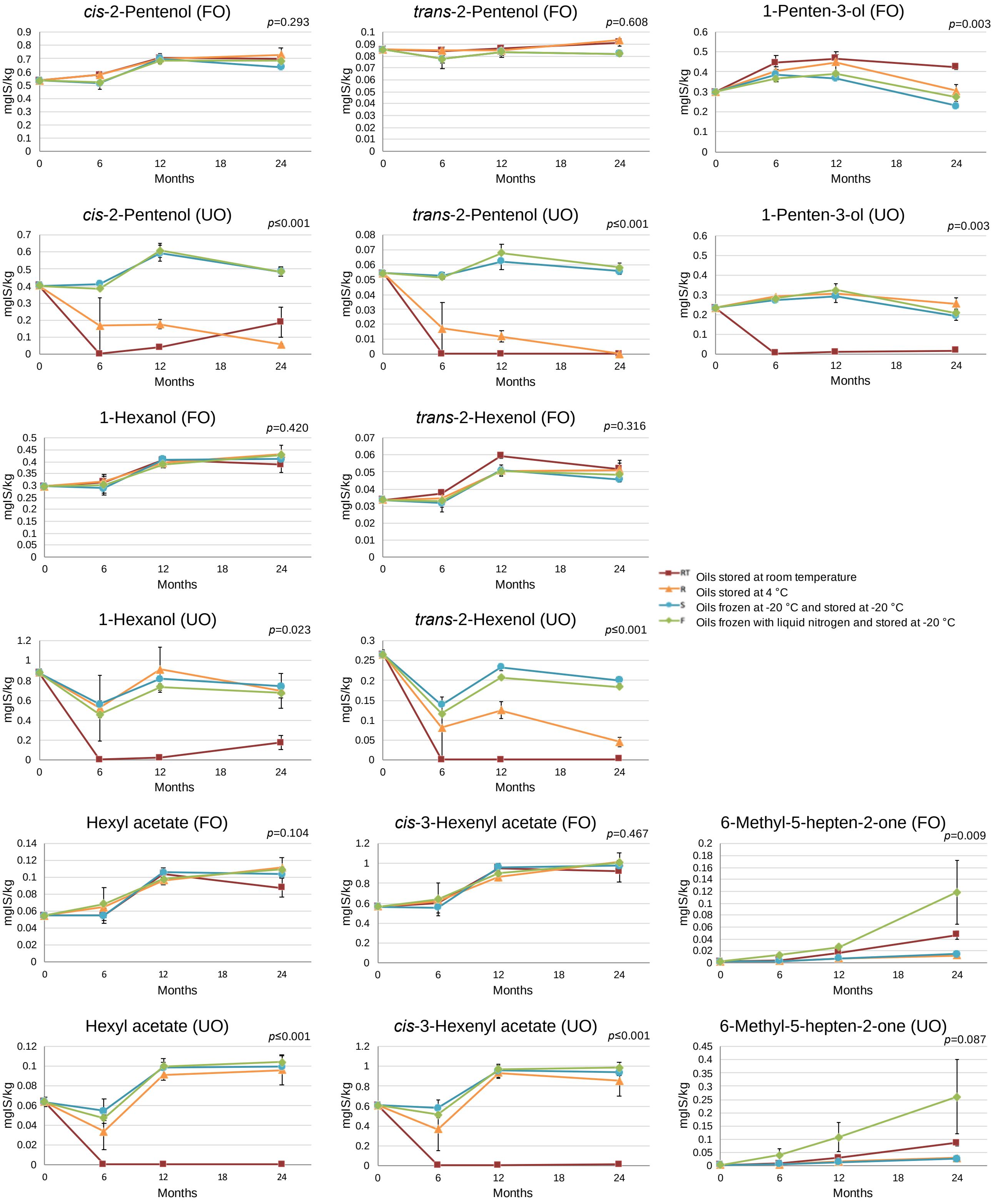

Supplement: Supplementary file 1 [file foods-12-01228-s001.zip › foods-2252486-supplementary.pdf]
